# Ultrafast and broadband photodetectors based on a perovskite/organic bulk heterojunction for large-dynamic-range imaging

**DOI:** 10.1038/s41377-020-0264-5

**Published:** 2020-03-03

**Authors:** Chenglong Li, Hailu Wang, Fang Wang, Tengfei Li, Mengjian Xu, Hao Wang, Zhen Wang, Xiaowei Zhan, Weida Hu, Liang Shen

**Affiliations:** 1 State Key Laboratory of Integrated Optoelectronics, College of Electronic Science and Engineering, Jilin University, 2699 Qianjin Street, Changchun, 130012 China; 2 State Key Laboratory of Infrared Physics, Shanghai Institute of Technical Physics, Chinese Academy of Sciences, 500 Yutian Road, Shanghai, 200083 China; 3 University of Chinese Academy of Sciences, Beijing, 100049 China; 4 Department of Materials Science and Engineering, College of Engineering, Key Laboratory of Polymer Chemistry and Physics of Ministry of Education, Peking University, Beijing, 100871 China

**Keywords:** Polymers, Optoelectronic devices and components, Imaging and sensing

## Abstract

Organic-inorganic hybrid perovskite (OIHP) photodetectors that simultaneously achieve an ultrafast response and high sensitivity in the near-infrared (NIR) region are prerequisites for expanding current monitoring, imaging, and optical communication capbilities. Herein, we demonstrate photodetectors constructed by OIHP and an organic bulk heterojunction (BHJ) consisting of a low-bandgap nonfullerene and polymer, which achieve broadband response spectra up to 1 μm with a highest external quantum efficiency of approximately 54% at 850 nm, an ultrafast response speed of 5.6 ns and a linear dynamic range (LDR) of 191 dB. High sensitivity, ultrafast speed and a large LDR are preeminent prerequisites for the practical application of photodetectors. Encouragingly, due to the high-dynamic-range imaging capacity, high-quality visible-NIR actual imaging is achieved by employing the OIHP photodetectors. We believe that state-of-the-art OIHP photodetectors can accelerate the translation of solution-processed photodetector applications from the laboratory to the imaging market.

## Perovskites peer into near-infrared

Novel photodetectors developed by researchers in China provide imaging in the near-infrared (NIR) region with record-breaking efficiency and speed. A new class of semiconducting materials called organic-inorganic hybrid perovskites (OIHPs) display excellent optical and electrical properties for thin-film solar cells, LEDs and light detectors. To expand their detection range to NIR, which is useful for biomedical applications, OIHPs can be combined with structures called organic bulk-heterojunctions (BHJs). Now, Weida Hu, Liang Shen and co-workers at Jilin University, Chinese Academy of Sciences and Peking University have designed new OIHP-BHJ photodetectors that efficiently detect a wide range of both visible and NIR radiation. Their prototype sensors have ultra-fast response times of just 5.6 nanoseconds and remain sensitive even in low brightness, suggesting that they could accelerate the movement of OIHP devices from lab tests to commercial imaging.

## Introduction

Serving as technical functional components for the translation of optical signals into electrical signals, photodetectors have received extensive attention and have been applied in various fields, including industrial production, military affairs, biochemical detection, optical communication, and scientific research^1–10^. The versatility and availability of photodetectors always depend on a few predominant factors: the photoresponse speed, sensitivity to lower brightness, detection band in which photodetectors can efficaciously detect light and dynamic range response^11–16^. Correspondingly, the key photodetector parameters that are to used to evaluate these performance factors are the response time or speed, spectral responsivity (*R*), noise current, external quantum efficiency (*EQE*), specific detectivity (D*) and linear dynamic range (LDR)^17–20^. Recently, the exploration of high-performance photodetectors has gradually become a research focus in the field of optoelectronics and high-quality imaging.

**Fig. 1:**
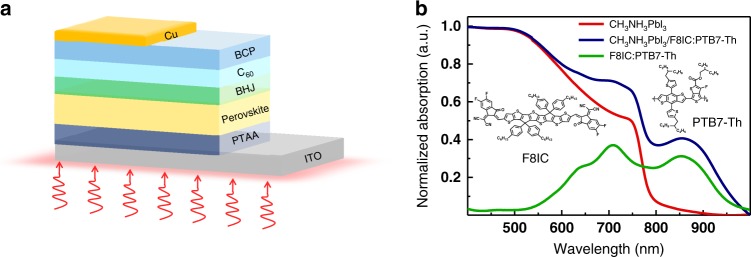
**a** Schematic device structure of the photodetectors. **b** Absorption spectra of different films by spin coating. Chemical structures of F8IC and PTB7-Th (inset).

Organic-inorganic hybrid perovskites (OIHPs) are emerging materials that have been progressively enabling new thin-film optoelectronics, including solar cells^21–27^, light-emitting diodes^28,29^ and photodetectors^14,16,30–36^. The extensive application of hybrid perovskites can be attributable to their excellent optical and electrical properties, including a direct bandgap, large absorption coefficient, high carrier mobility, and low trap density^37–40^. Therefore, OIHP photodetectors have demonstrated high *R*, high D*, an ultrafast response speed and a high LDR when combined with device structure engineering^11,18,41^. However, the detection range of MAPbI_3_ (either polycrystalline films or thin single crystals) is limited to the wavelength region below 820 nm and does not cover the near-infrared (NIR) range, which severely limits its application, especially in biomedical imaging. To overcome this problem, an advantageous strategy has been demonstrated: combining OIHP and an organic bulk heterojunction (BHJ) consisting of donor-acceptor materials with light absorption in the NIR region^16,35,36,42^. Shen et al. reported a composite photodetector based on MAPbI_3_ and PDPPTDTPT/PCBM, which exhibited a wider detection wavelength extending to 950 nm with a 5 ns ultrafast response time^16^. This work provided an effective way of achieving both a wider and faster response for next-generation photodetectors. However, the sole flaw of the photodetectors was that the *EQE* value in the NIR region failed to reach a similar value to that in the UV-visible range, which resulted from the weak NIR absorption of the low-bandgap polymer and a mismatched energy level alignment at the interface between the OIHP and BHJ layers. Wang et al. reported photodetectors based on MAPbI_3_ and PDPP3T/PC_71_BM BHJ, achieving a slightly higher *EQE* of 40% in the NIR region. However, the achieved response time on the order of microseconds cannot easily meet the application requirements^36^. Recently, Wu et al. demonstrated a broadband photodetector with an *EQE* of 70% in the NIR region by coating PTB7-Th:IEICO-4F on MAPbI_3_^35^. However, the photodetectors did not display an inspiring performance in terms of a lower noise current and an extremely fast response time. State-of-the-art OIHP broadband photodetectors should have a high *EQE* value in the NIR region, high sensitivity and an ultrafast response speed. However, no such results have been reported to date. Compared with previously reported NIR materials such as PDPPTDTPT, PDPP3T, and IEICO-4F, a fused-ring electron acceptor named F8IC with a lower bandgap and higher electron mobility has been successfully synthesized^43^. F8IC exhibits an extremely low bandgap of 1.43 eV, which matches well with the energy levels of the polymer donor PTB7-Th (highest occupied molecular orbital (HOMO) energy level of −5.20 eV, Lowest Unoccupied Molecular Orbital (LUMO) energy level of −3.59 eV) to constitute the organic BHJ. The structural formulas of the two materials are shown in the inset of Fig. 1b. Polymer solar cells (PSCs) based on a PTB7-Th:F8IC blend have shown a power conversion efficiency (PCE) of 10.9% with a high *EQE* extending into the NIR region^43^. Furthermore, the higher electron mobility of F8IC is ten times higher than that of IEICO-4F^44,45^, enabling a faster response speed for hybrid photodetectors. In addition, the absorption of fullerenes in the NIR region is very weak, resulting in a low NIR response. The nonfullerene F8IC has a stronger NIR absorption and a better energy level alignment to match the perovskite layers than a fullerene system. F8IC generates excitons, which can be dissociated into electrons and holes under NIR light excitation. Photogenerated electrons will directly transfer to C_60_ and then be collected by the cathodes, while NIR photogenerated holes can be transported to perovskite through PTB7-Th and finally arrive at the anodes through the PTAA layer. Herein, an organic BHJ consisting of PTB7-Th:F8IC is introduced into OIHP to structure photodetectors, yielding a broad *EQE* covering 1000 nm with a peak of 54% in the NIR region. In addition, the photodetectors exhibit a high D* of over 2.3 × 10^11^ Jones (cm Hz^1/2^ W^−1^) at 870 nm and an ultrafast response time of 5.6 ns. To the best of our knowledge, this is the top-ranking level response speed reported for NIR OIHP photodetectors. More importantly, the broadband photodetector has practical application ability, which is demonstrated with high-quality imaging in both the visible and NIR ranges due to its high-dynamic-range imaging capacity. We believe that state-of-the-art hybrid perovskite photodetectors can provide a powerful supplement for inorganic counterparts to meet more energetic requirements.

## Results and discussion

Fig. 1a shows a schematic structure of the OIHP/BHJ photodetectors, which consist of indium tin oxide (ITO)/poly(bis(4-phenyl) (2,4,6-trimethylphenyl) amine (PTAA)/CH_3_NH_3_PbI_3_ (MAPbI_3_)/F8IC:PTB7-Th/ C_60_/ 2,9-dimethyl-4,7- diphenyl-1,10-phenanthroline (BCP)/copper (Cu). The OIHP polycrystalline films were grown equably on PTAA-modified ITO conductive glass, and the F8IC:PTB7-Th film acted as an NIR light photosensitive layer on the OIHP active layer. Fig. 1b shows the absorption spectra of pure MAPbI_3_, F8IC:PTB7-Th and MAPbI_3_/F8IC:PTB7-Th composite films. The BHJ film reveals a dominant absorption band covering 550 nm to 1000 nm with a visible peak at 710 nm and an NIR peak at 850 nm. Benefitting from the NIR complementation, the MAPbI_3_/F8IC:PTB7-Th composite film exhibits a broader absorption in the region of 400–1000 nm than pure MAPbI_3_ (400–780 nm), confirming the theoretical feasibility of fabricating UV-vis-NIR broadband photodetectors.

**Fig. 2:**
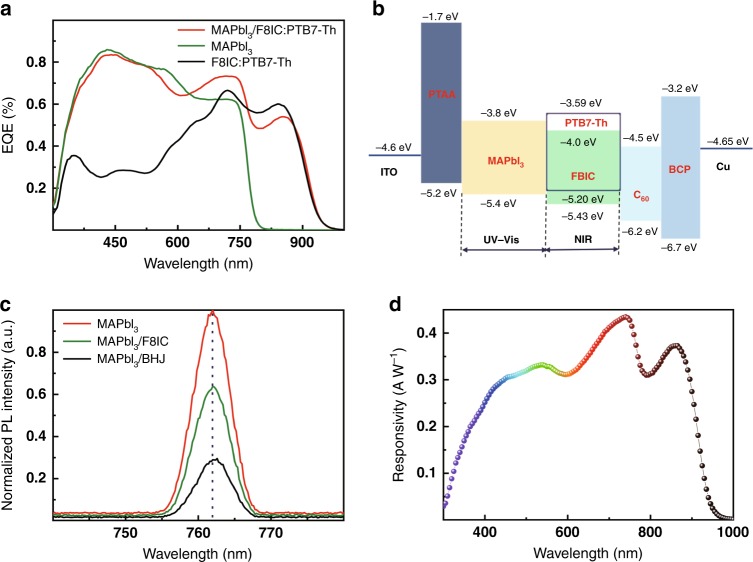
**a** *EQE* curves of the perovskite/organic BHJ hybrid photodetectors, pure perovskite and pure BHJ devices at zero bias and 70 Hz. **b** Band energy level alignment of the perovskite/organic BHJ hybrid photodetectors in this study. **c** PL of the film of MAPbI_3_ bound to different materials. **d** Responsivity of the corresponding perovskite/organic BHJ hybrid broadband photodetector.

Fig. 2a shows the *EQE* spectra of perovskite/BHJ photodetectors measured at zero bias. The response spectrum of the photodetectors can be extended to 1000 nm, which is in accordance with the composite absorption range. The photoresponse provided by the BHJ is ideally complementary to that of halide perovskites, significantly enhancing the *EQE* spectra from 600 to 1000 nm for the MAPbI_3_ photodetectors. Encouragingly, the *EQE* value of 54% at 850 nm for the perovskite/BHJ photodetectors provides direct evidence that the charge carriers generated in the NIR region can be sufficiently collected by the electrodes. Fig. 2b displays the energy level diagram of the perovskite/BHJ photodetectors. In principle, a strictly matched energy level for electron and hole transport can enable good device performance. The NIR photogenerated holes may be extracted completely by MAPbI_3_ due to the proper alignment of the energy level. To deeply investigate the mechanism of the charge transport in the interface between the organic BHJ and perovskite, photoluminescence (PL) spectra of the films were measured as shown in Fig. 2c using excitation light at 380 nm. The PL intensity of F8IC:PTB7-Th/MAPbI_3_ decreases sharply compared with that of F8IC/MAPbI_3_, indicating that more electrons are extracted and transported through the films when mixing F8IC and PTB7-Th as the donor-acceptor BHJ^46,47^. The luminescence characteristics provide direct evidence that a more brilliant interface state and interlayer coupling is achieved between the MAPbI_3_ and F8IC:PTB7-Th layer and that the carriers can transfer effectively at the interface. The NIR photogenerated electrons will be collected by the cathode through the C_60_ electron transport layer, and the NIR photogenerated holes can be transported via perovskite to the ITO anode owing to the high hole mobility of MAPbI_3_. On the other hand, the UV-visible photogenerated electrons in the perovskite layer can be transported to the cathode through the organic BHJ layer and C_60_, with the photogenerated holes collected directly by the anode. This working photodetector mechanism ensures the effective detection of both UV-visible and NIR light, which provides theoretical guidance for broadband photodetection.

**Fig. 3:**
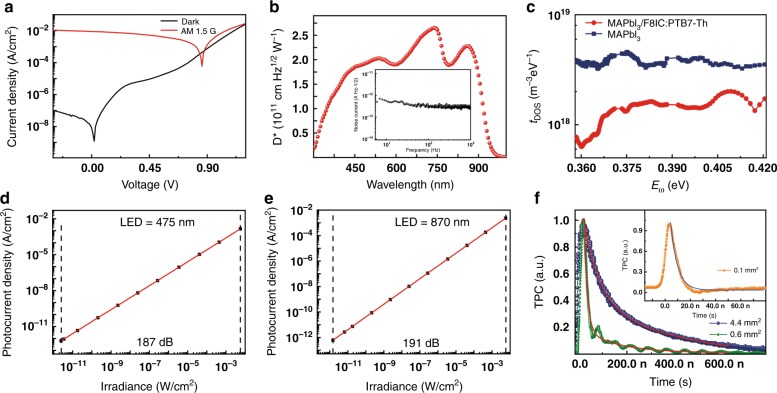
**a** Photocurrent density (under air mass 1.5 G illumination) and dark current density curves of the broadband photodetectors. **b** Specific detectivity D* of the broadband photodetector at various light wavelengths under a bias voltage of −0.1 V. **c** Trap density of states curves of the perovskite/BHJ hybrid photodetectors. **d** Linear dynamic range of the broadband photodetectors under 475-nm LED illumination with various light intensities. The solid line represents linear fitting to the data. **e** LDR under 870-nm LED illumination. **f** Transient photocurrent curves of the broadband photodetector with device areas of 0.6 and 4.4 mm^2^. Inset: TPC curve of the ultrafast photodetectors with a smaller area of 0.1 mm^2^.

Fig. 2d indicates the responsivity (*R*) curves of the photodetectors, which can be expressed from the *EQE* curve according to the equation

1 $$R = EQE \times q\lambda /hc$$

where *h* is Plank’s constant, *c* is the velocity of light, *q* is the absolute value of the electron charge, and *λ* is the light wavelength. The OIHP photodetectors exhibit a wide range from 300 nm to 1000 nm, and the peak in the NIR region can reach up to 0.37 AW^−1^ (870 nm) accompanied by approximately 0.43 AW^−1^ in the visible spectrum. Fig. 3a displays the dark current and photocurrent (under air mass 1.5 G illumination) density curve of the perovskite/organic BHJ photodetectors for voltages ranging between −0.3 and 1.2 V. The dark current density is as low as 3.4 × 10^−8^ A cm^−2^ at −0.1 V, suggesting a relatively low noise current and high sensitivity. As shown in Fig. 3b (inset), the noise current of the perovskite/BHJ photodetector is 3.6 × 10^−13^ A Hz^−1^ at 70 Hz (corresponding to the frequency of the *EQE* measurement). The actual detection capability of the photodetectors to monitor weak signals can be expressed by the specific detectivity (D*) determined by the responsivity and noise of the photodetectors, which can be calculated by the following equations:^48^

2 $${\it{D}}^ \ast = \frac{{\sqrt {AB} }}{{NEP}}\left( {cm\;Hz^{ - 1/2}{\it{W}}^{ - 1}\;or\;Jones} \right)$$

3 $${\mathrm{NEP = }}\frac{{i_n}}{R}\left( {{\mathrm{WHz}}^{ - {\mathrm{1/2}}}} \right)$$

where *A* is the active layer area, *i*_n_ is the noise current, *B* is the bandwidth and NEP is the noise equivalent power. As shown in Fig. 3b, the hybrid perovskite/organic BHJ photodetectors exhibit a D* of 2.3 × 10^11^ Jones (cm Hz^1/2^ W^−1^) in the 870 nm NIR region, indicating the high performance of the fabricated broadband photodetector. Fig. 3c displays the trap density of states (*t*_DOS_) obtained by thermal admittance spectroscopy of two different devices^48,49^. The pure perovskite photodetectors possess a relatively large density of defect states of 3 × 10^18^ to 6 × 10^18^ m^−3^ eV^−1^ without the BHJ. However, the hybrid perovskite/BHJ device exhibits reduction in *t*_DOS_ of nearly one order of magnitude. This result can perfectly verifies the low trap density of our broadband photodetectors and is important for a fast photodetector response.

In practical photodetector applications, especially in an imaging system, a constant responsivity covering a large range of light intensities is critically significant for extracting the specific intensity of the detected light from the corresponding photocurrent. The linear weak-light response range is always characterized by the linear dynamic range (LDR), defined as an optical power margin within which the output photocurrent is linearly proportional to optical signal input:

4 $${\mathrm{LDR = 20}} \,\times\, {\mathrm{log}}\frac{{P_{{\mathrm{max}}}}}{{P_{{\mathrm{min}}}}}$$

where *P*_max_ and *P*_min_ are the upper and lower limits of the optical power in a particular range. Notably, a sufficiently large LDR means maintaining a constant responsivity from strong light to weak light conditions and is a precondition for weak light sensing. Fig. 3d, e show the LDR of photodetectors illuminated by LEDs with different wavelengths (475 nm and 870 nm). It can be clearly observed that the photocurrent density increases linearly for a dynamic light intensity ranging from 2.5 pW cm^−2^ to 5.6 mW cm^−2^ with the 475 nm LED and 1.5 pW cm^−2^ to 5.6 mW cm^−2^ with the 870 nm LED. This result corresponds to LDR values of 187 dB (475 nm) and 191 dB (870 nm), respectively, demonstrating the universal applicability of our device to various light sources, which is an essential prerequisite in a high-quality imaging system. Such a linear response may result from the excellent carrier transport property of the perovskite with the BHJ and the low electron trap density in the whole photodetector.

Finally, the response speed of this broadband photodetector is discussed in detail to completely characterize its performance parameters. The light response speed is a significant core performance parameter that determines the quality of photodetectors. A general method that is used to meausre an ultrafast response speed is the transient photocurrent (TPC)^12,14^. The photodetectors collect the pulsed light signal emitted by a pulsed laser, and then the photogenerated carriers can be driven by a built-in potential field or external voltage bias applied to the respective electrodes. Therefore, the response speed can be defined as the photocurrent decay time from the peak down to approximately 1/e after a single exponential fit to the TPC curve. Excited by a pulsed laser at a wavelength of 850 nm, the normalized TPC curves of the photodetectors with different active areas are shown in Fig. 3f. The response time of the perovskite/organic BHJ photodetectors is calculated to be 145 ns for a relatively large active area of 4.4 mm^2^ and 19 ns for small devices with an area of 0.6 mm^2^. This indicates that the response time is limited by the resistance-capacitance (RC) time constant, which is typically mixed with the carrier transit time, resulting in a deviation of the actual response speed.

5 $$f_{ - {\mathrm{3dB}}}^{ - 2} = \left( {\frac{{{\mathrm{3}}{\mathrm{.5}}}}{{{\mathrm{2}}\pi t_{{\mathrm{tr}}}}}} \right)^{ - 2} \,+\, \left( {\frac{1}{{{\mathrm{2}}\pi {\mathrm{RC}}}}} \right)^{ - 2}$$

where *t*_tr_ is the carrier transit time and the RC time is the resistance-capacitance time constant of the circuit. In Fig. 3f (inset), to ensure the operation of the device, we reduced the area of the best performing device to 0.1 mm^2^ and obtained an ultrafast speed of 5.6 ns at zero bias. This ultrafast response is also commendable in the field of infrared photodetectors^50^. The outstanding response speed is due to a variety of causes, including the low trap density of the active layers, higher carrier mobility of the transport layers and suppression of the RC time. Herein, the RC time and transit time of the whole device are estimated based on the reported carrier mobility of the material and the actual thickness of each layer of the photodetectors. The calculation process is described in the supporting information, which obtains an RC time of 2.3 ns as calculated by Eqs. 1 and 2 in the SI, indicating the veracity of the practically measured response speed of 5.6 ns by the TPC method. The response time of the perovskite/organic BHJ photodetectors with an active area of 4.4 mm^2^ was also tested by the standard square wave method, and a rise/fall time of ~35/20 μs was obtained, as shown in Supplementary Fig. S2.

**Fig. 4:**
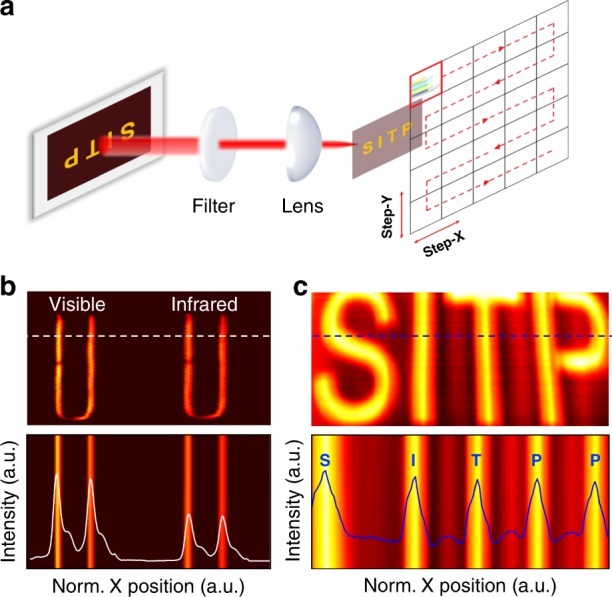
**a** Schematic of the image scanning system and actual imaging for the OIHP photodetector. **b** Visible and NIR (with an 830-nm long-pass filter) imaging results of the heat coil. **c** Imaging of SITP (an abbreviation of Shanghai Institute of Technology and Physics) letter graphics under LED illumination. The white and blue lines in the figure represent the normalized photocurrent signal intensity.

To further demonstrate the practical feasibility of the high-performance broadband photodetectors, we explored the functional role of the devices in imaging technology^51–54^. Generally, the photosensitive component in mature imaging fields such as digital cameras is a charge-coupled device (CCD), which can sense light and convert the image into digital form. From a functional point of view, the CCD components can be replaced with perovskite/BHJ broadband photodetectors to a certain extent. Herein, we adopt a high-performance imaging experiment to verify the high detectivity, broadband detection and ultrafast response of the OHIP photodetectors. The broadband photodetector scans the imaging target in two dimensions via the driving of the two-dimensional turntable, and the photocurrent of each pixel is recorded, avoiding the complicated structure of array imaging sensors. Considering that the response of the perovskite/BHJ photodetectors covers a wide wavelength range, including the NIR region, a blackbody-like, radiant heat source is employed as an imaging target. Detailed imaging devices and processes are shown in Fig. 4a. The light of the imaging target passes through the optical system, which can be focused on the sample by lenses. An 830-nm long-pass filter is placed in front of the lens to eliminate the effects of the band before 830 nm when NIR imaging alone is required. The current generated by the sample flows through resistance, and the voltage signal across the resistance can be output by the amplifier. The corresponding image is transmitted from the output voltage of each pixel to the computer via the gray code operator^55^. Fig. 4b shows the visible and NIR imaging results of the radiation source. The profile of the heat coil can be clearly captured in both the visible and NIR bands, and the change in the photocurrent intensity of the corresponding graphical position displays an obvious difference between the visible and NIR regions. This means that our device can completely distinguish the target in the environment and restore the display to a certain extent, highlighting the visible-NIR dual-waveband recognition imaging ability of perovskite broadband photodetectors. Moreover, for more realistic scenarios, we designed SITP letter graphics on an LED screen to verify the imaging capability of perovskite photodetectors for a more complex target. Using the same method, a high-quality image is achieved based on the corresponding voltage and position, which is illustrated in Fig. 4c. The pattern of letters with sharp boundaries is obtained, and simultaneously, the consistency of the position or distance between the images and objects proves the high fidelity of our photodetectors in the field of imaging. Here, we attribute the results to the high-dynamic-range imaging (HDRI) capability of the photodetectors, which is a crucial feature for high-quality imaging. When the high brightness and shadow areas of an actual image object have sharp contrast, image sensors with an inferior HDRI capability tend to obscure low-brightness targets with noise. The high brightness of a target will result in corresponding pixel saturation and even overflow, leading to an unclear image. In this study, the optical power exhibits a gradient from the background to the pattern body, meaning that the three parts of the “background - pattern edge - pattern body” have different optical powers. By successfully capturing this difference and demonstrating a significant imaging result, the preeminent HDRI capability of the perovskite photodetector is validated. The intensities of white light signals are clearly distinguished by the contrasting colors in Fig. 4c. The dynamic photocurrent intensity along the dotted line reveals the weak light extraction ability of the broadband photodetector and the realization of large-dynamic-range imaging. Benefiting from the excellent intrinsic performance of perovskite photodetectors with high sensitivity, low noise, and a large LDR, the difficulty of complex algorithm processing for high-quality imaging is greatly reduced. Moreover, relevant studies may further improve the imaging capabilities by adjusting the device size, adding pixels, and designing an array geometry.

In summary, we have demonstrated solution-processed UV-vis-NIR broadband photodetectors based on MAPbI_3_ and an organic BHJ, achieving broadband response spectra up to 1000 nm with an *EQE* peak of 54% in the NIR region. The response time of 5.6 ns represents the fastest speed of perovskite broadband photodetectors. Importantly, the large LDR of the photodetectors contributes to high-dynamic-range imaging, and the superior processing capacity of the photodetectors for actual imaging applications has practical application value in many fields. We believe that our results can inspire more fundamental or extended studies in the future.

## Materials and methods

### Materials

PbI_2_ (>99.99%), MAI, and PTAA were purchased from Xi’an p-OLED (Xi’an China). Bathocuproine (BCP) and fullerene (C_60_) were obtained from Lumtec (Taiwan, China). PTB7-Th was purchased from A One Material (Taiwan, China). These materials were used as received.

### Device fabrication and measurements

All of the solutions were prepared at room temperature in an air environment. The PTAA was dissolved in toluene at a concentration of 2 mg mL^−1^. To fabricate the perovskite film, we adopted the common one-step method: PbI_2_ and MAI molar ratio 1:1 was dissolved in a 9:1 DMF (dimethylformamide):DMSO (dimethyl sulfoxide) mixed solution to make a MAPbI_3_ prosomatic solution with a concentration of 0.75 mol mL^−1^, and the solution was magnetically stirred at 60 °C until completely dissolved. For the organic donor-acceptor BHJ layer, F8IC:PTB7-Th were mixed and dissolved in chlorobenzene (CB) in a 1:1 proportion (the concentration of the mixed solution was 10 mg/mL). Then, the mixed solution was magnetically stirred at room temperature for 6–10 h.

The macrostructure of our photodetector is shown in Fig. 1b. The ITO substrates were ultrasonically cleaned by acetone and ethanol for 20 min and treated with UV ozone for 10 min. Then, a thin PTAA layer was simply spin-coated at 4000 r.p.m. for 40 s on the ITO surface, and the substrates were baked at 100 °C for 20 min. The MAPbI_3_ solution was deposited onto the prepared HTL films at 4000 r.p.m. for 40 s, cleaned by anhydrous toluene, and then annealed at 100 °C for 1 h to obtain a perovskite active layer. Subsequently, the hybrid F8IC:PTB7-Th solution was spin-coated on the perovskite film at 2000 rpm and annealed at 80 °C for 20 min. C_60_ (varying the thickness as required) and BCP (7 nm) were thermally evaporated by vapor deposition onto the organic BHJ layer. The evaporation rates was approximately 0.03 nm/s for C_60_, 0.03 nm/s for BCP, and 0.06 nm/s for Cu. Finally, the devices were completed by depositing a 100-nm Cu electrode. Thus far, a device with an effective area of 0.044 cm^−2^ has been completely manufactured.

A Shimadzu UV-1700 Pharma Spec UV spectrophotometer was used to measure the absorption spectra of the OIHP photodetectors. A Shimadzu RF 5301 fluorescence spectrophotometer was used to obtain the PL spectra of our devices. The external quantum efficiencies were measured by a Crowntech Q test Station 1000 AD measurement system. The *J*–*V* curves of our photodetectors in the dark and under illumination were obtained by a Keithley 2601 source meter. The incident light was provided by AM 1.5 G solar illumination with an Oriel 300 W solar simulator intensity of 100 mW cm^−2^. The noise current was analyzed by a ProPlus 9812D wafer-level 1/f noise characterization system. The measurement of the response speed was carried out by the transient photocurrent (TPC) method: A gold probe was used in the TPC measurement to minimize the external impact caused by the poor transmission of the electrical signals. The area of the pulsed beam was 10 mm^2^, larger than the device area. The rated power of the Ti:Sapphire femtosecond laser is 1 W, with a 1 kHz repetition rate. The pulsed Ti-sapphire femtosecond laser was prepared as the optical source, and the luminescence wavelength was set to 850 nm, with frequency doubling and a pulse duration of 150 fs. The photodetectors collected the pulsed light signal emitted by the pulsed laser, and then a 1 GHz oscilloscope with a 5 GHz sampling rate recorded the current pulse and generated the corresponding TPC curve. The response speed can be defined as the photocurrent decay time from the peak down to approximately 1/e after a single exponential fit to the TPC curve. To minimize the influence of the inductance of the whole circuit, the cables connecting the device and oscilloscope should be as short as possible and connected with a fast (6 GHz) bayonet Neill-Concelman connector. In addition, the measurement environment was kept at room temperature with no light sources other than the lasers. In addition, the environmental conditions of the photodetector performance measurement were room temperature (25 °C), 20% humidity, and normal pressure. The imaging process was generally conducted at room temperature (25 °C), a humidity of >45%, and an imaging time of >1 h.

## Supplementary information

Supplementary Information for Ultrafast and Broadband Photodetectors Based on a Perovskite/Organic Bulk Heterojunction for Large-Dynamic-Range Imaging
