# Supplementary material for: Ultrafast and broadband photodetectors based on a perovskite/organic bulk heterojunction for large-dynamic-range imaging
Source: Light Sci Appl. 2020 Mar 3;9:31. doi: 10.1038/s41377-020-0264-5 (PMC7054320; doi:10.1038/s41377-020-0264-5)
Supplement: Supplementary file 1 — Supplementary Information for Ultrafast and Broadband Photodetectors Based on a Perovskite/Organic Bulk Heterojunction for Large-Dynamic-Range Imaging [file 41377_2020_264_MOESM1_ESM.docx]

**Supplementary Information for**

**Ultrafast and Broadband Photodetectors Based on a Perovskite/Organic Bulk Heterojunction for Large-Dynamic-Range Imaging**

*Chenglong Li,^1,#^ Hailu Wang, ^2,3,#^ Fang Wang, ^2,3^* Tengfei Li, ^4^ Mengjian Xu, ^2^ Hao Wang, ^2,3^ Zhen Wang, ^2,3^ Xiaowei Zhan, ^4^ Weida Hu,^2,3^* Liang Shen ^1^**

**Calculation of the RC time constant**

The perovskite photodetector structure can be divided into several layers between the ITO and Cu electrode. According to the definition of capacitance in series, the total capacitances are composed five branches expressed by formula (1) and (2)^1, 2^:

$\frac{\text{1}}{\text{C}}\text{=}\frac{\text{1}}{\text{C}_{\text{PTAA}}}\text{+}\frac{\text{1}}{\text{C}_{\text{perovskite}}}\text{+}\frac{\text{1}}{\text{C}_{\text{BHJ}}}\text{+}\frac{\text{1}}{\text{C}_{\text{C60}}}\text{+}\frac{\text{1}}{\text{C}_{\text{BCP}}}$ (1)

$\text{C = A}\text{ε}_{\text{0}}\text{ε}_{\text{r}}\text{/D}$ (2)

where *D* is the film thickness. The *C* can be calculated as follows:

*C*_PTAA_ = 1.40 nF, *C*_perovskite_ = 0.215 nF, *C*_BHJ_ = 0.08 nF, *C*_C60_ = 0.36 nF, *C*_BCP_ = 0.95 nF.

*C* = 0.046 nF; *RC* = 2.3 ns.

**Calculation of the transit time**

The travel of carriers from one electrode to the opposite electrode under the action of a built-in electric field or bias voltage was defined as the transit time, which could be determined by the equations:

$\text{ t= }\frac{\text{D}^{\text{2}}}{\text{μV}}$ (3)

$\text{V = }\frac{\text{Q}}{\text{C}}\text{ = }\frac{\text{QD}}{\text{A}\text{ε}_{\text{0}}\text{ε}_{\text{r}}}$ (4)

Where *t* is the transit time, *μ* is the carrier mobility, *V* is the partial voltage of each layer, and *D* is the film thickness. It can be seen that *V* is proportional to *D/ε_r_*. *V_oc_* = 0.88 V. By calculating the partial voltage in term of the formula, the estimated *V* of each layer is as follows:

*V*_PTAA_ = 0.019 V, *V*_Perovskite_ = 0.46 V, *V*_BHJ_ = 0.11 V, *V*_C60_ = 0.22 V, *V*_BCP_ = 0.08 V. *μ*_PTAA_ ≈ 5×10^-3^ cm^2^ V^-1^ s^-1^, *μ*_Perovskite_ ≈ 30 cm^2^ V^-1^ s^-1^, *μ*_BHJ_ ≈ 10^-3^-10^-2^ cm^2^ V^-1^ s^-1^, *μ*_C60_ ≈ 10^-1^-10^-2^ cm^2^ V^-1^ s^-1^, *μ*_BCP_ ≈ 2×10^-2^ cm^2^ V^-1^ s^-1^ ^1-8^.

The estimated results of the transit time by optimal mobility are as follows:

*t*_PTAA_ ≈ 0.10 ns, *t*_perovskite_ ≈ 0.065 ns, *t*_BHJ_ ≈ 0.13 ns, *t*_C60_ ≈ 0.14 ns, *t*_BCP_ ≈ 0.31 ns

**Response time**

**
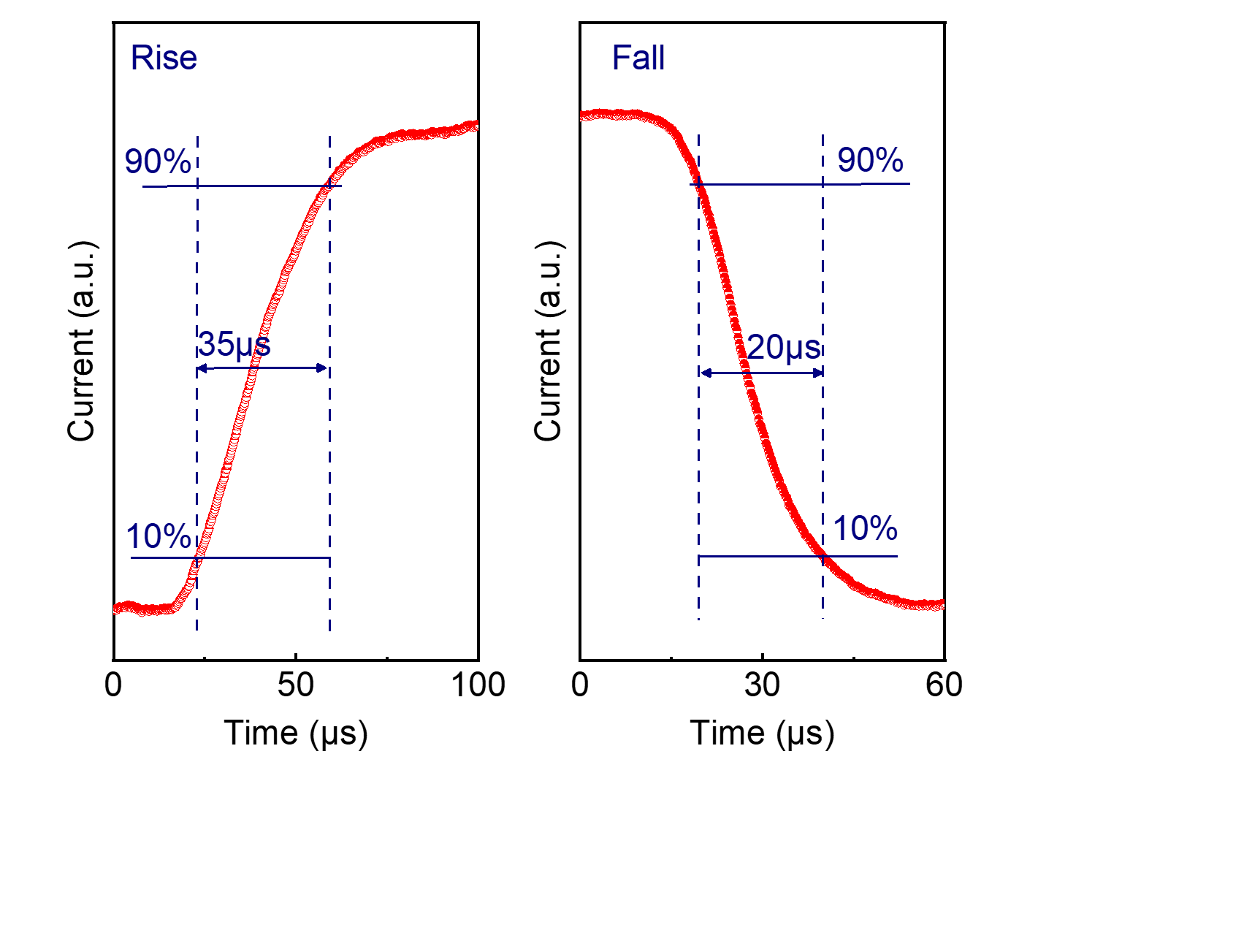
**

**Fig. S1.** Time-resolved photoresponse of the perovskite/organic bulk-heterojunction photodetector at *V*_ds_ = 1V under 830 nm laser illumination by standard square wave method.

In general, the rise/fall time is defined as the time for the photocurrent to increase/decrease from 10%/90% to 90%/10% of the peak when photocurrent arrives at a stable state after accepting/removing illumination.

**Linear dynamic range**


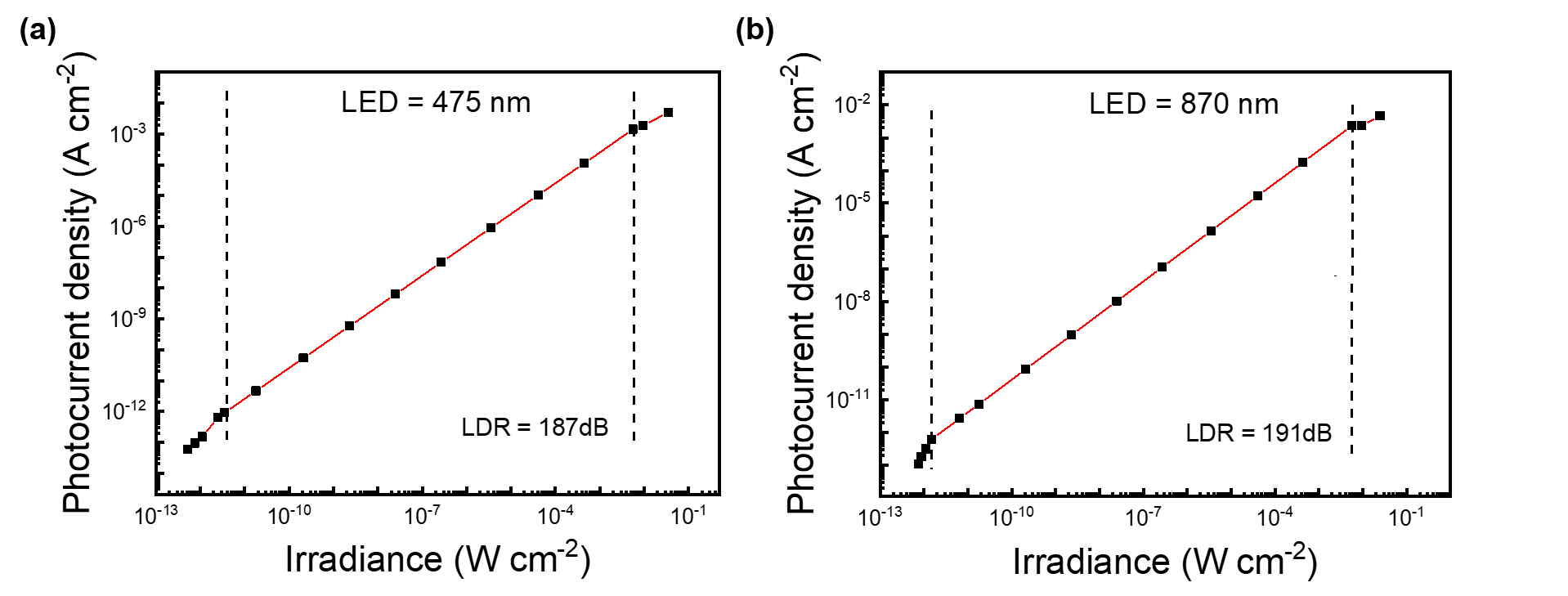


**Fig. S2.** Relationship between photocurrent density and irradiance under a LED illumination. a) 475 nm; b) 870 nm of various power intensities. LDR can be extracted from the linear region (between two black dotted lines).

**Optimization of photodetectors**

For the broadband photodetectors with different C_60_ thicknesses, the *I*-*V* curves under dark and simulated AM 1.5G solar irradiation were characterized.


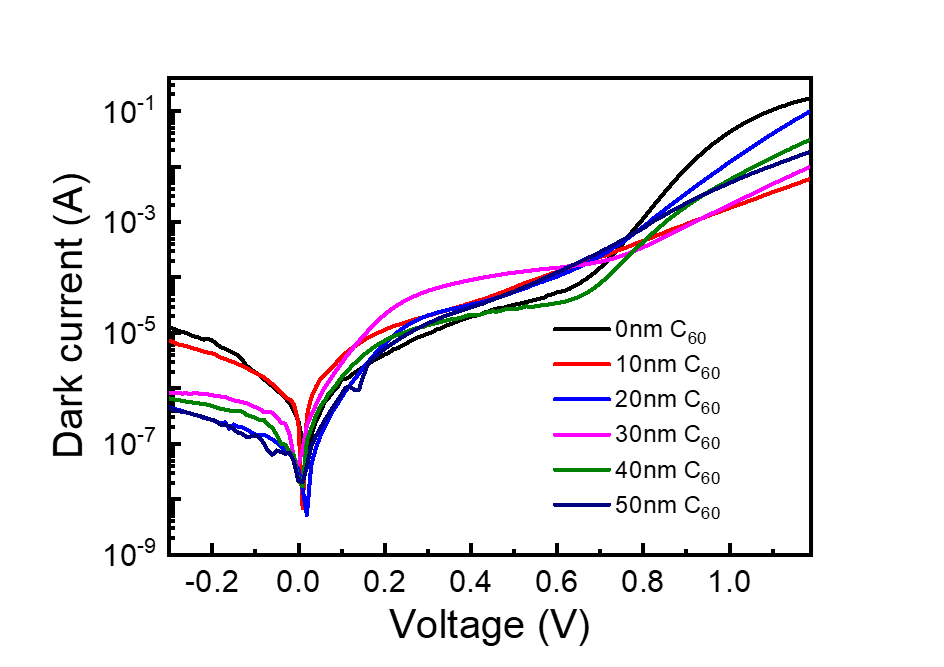


**Fig. S3.** *I*-*V* curves of photodetectors with different thicknesses of C_60_ in the dark.


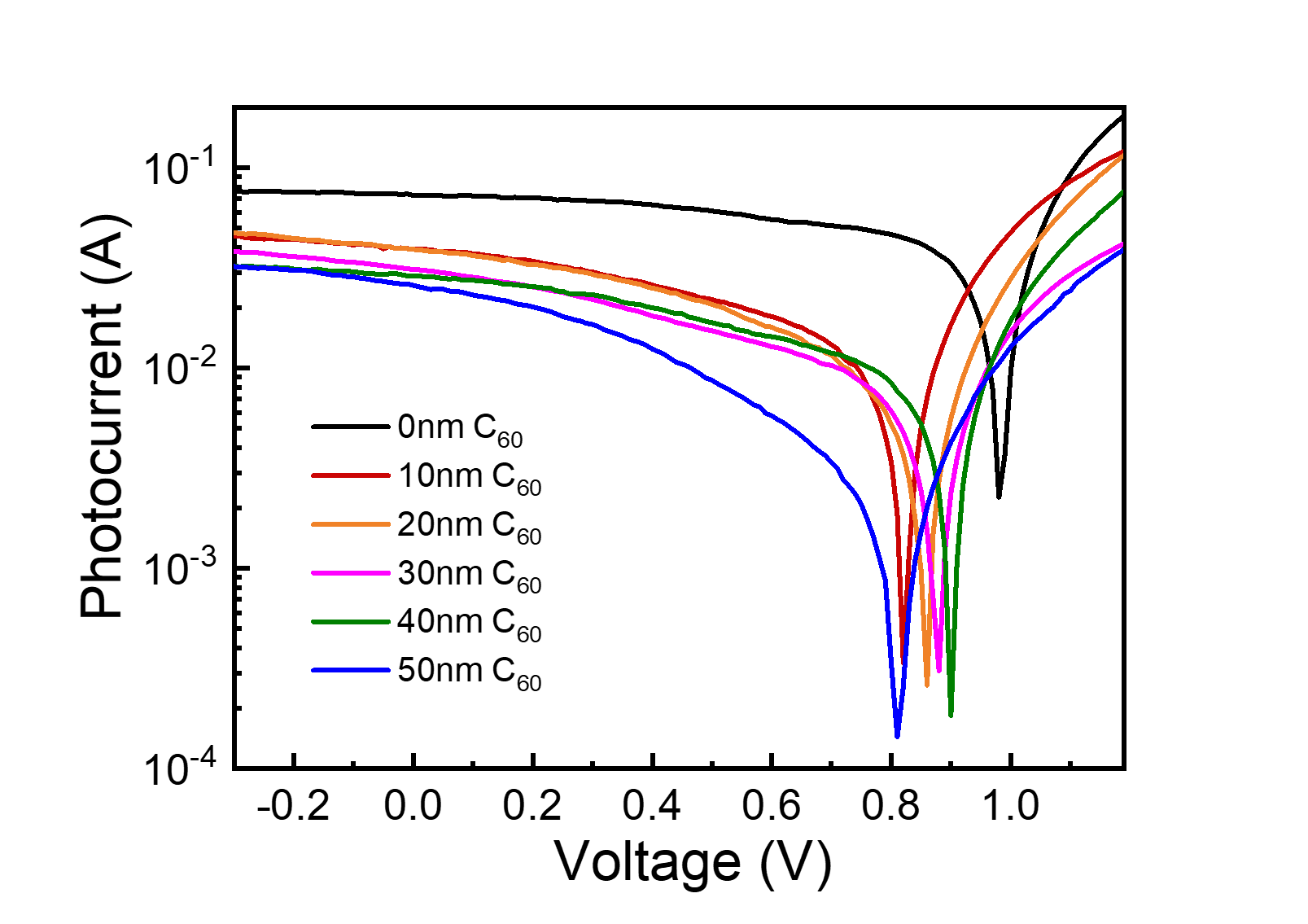


**Fig. S4.** *I*-*V* curves of photodetectors with different thicknesses of C_60_ under the simulated AM 1.5G solar irradiation.

It is more convenient to distinguish devices by the ratio of light and dark current *(I*_light_*/I*_dark_*)*, as shown in **Fig. S5**.

**
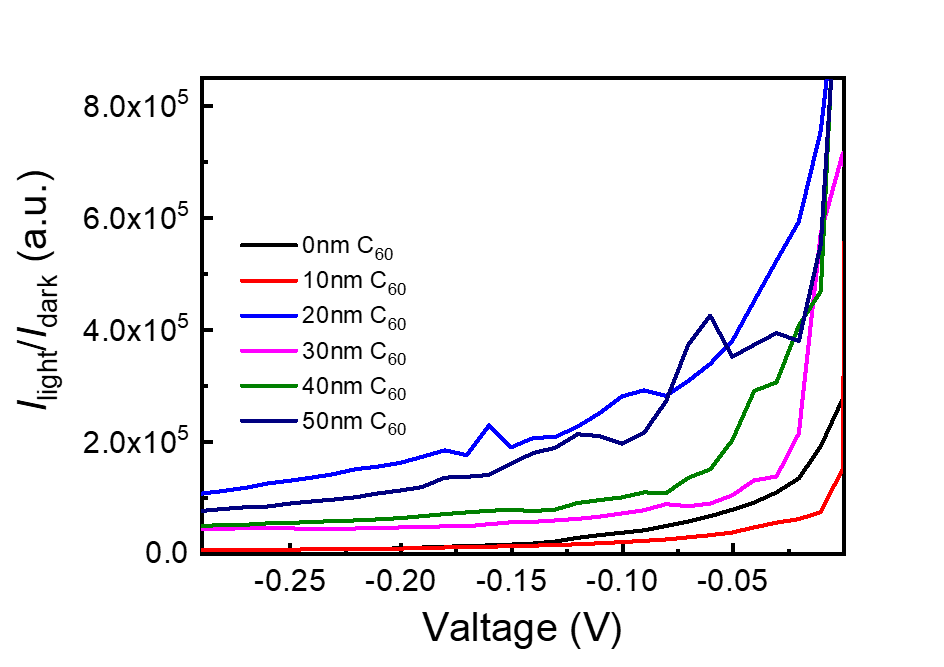
**

**Fig. S5.** *I*_light_/*I*_dark_ of photodetectors with different thicknesses of C_60_.

**
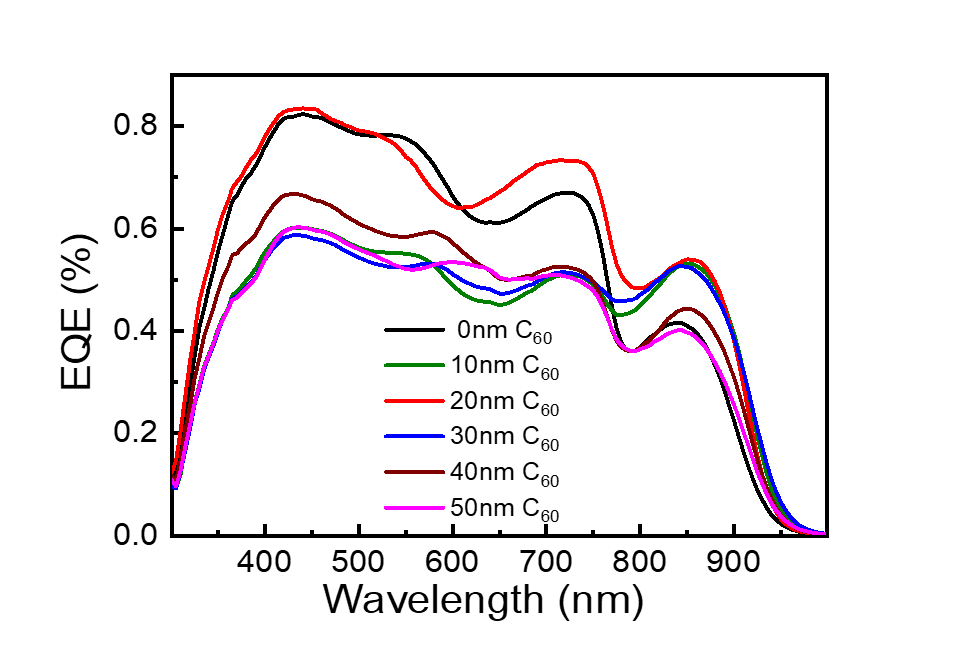
**

**Fig. S6.** *EQE* values of the photodetectors with different thicknesses of C_60_.

It can be seen that the broadband photodetectors with 20nm C_60_ showed the best performance.

**Table S1.** Parameter values of devices with different thicknesses of C_60_.

| **Thickness of C_60_ (nm)** | ***I*_dark_ at**  **-0.1V** | ***I*_light_ at**  **-0.1V** | **Visible *EQE* at 440 nm** | **NIR *EQE* at 850 nm** |
| --- | --- | --- | --- | --- |
| 0 | 1.92×10^-8^ A | 7.47×10^-4^ A | 82% | 41% |
| 10 | 1.94×10^-8^ A | 4.13×10^-4^ A | 60% | 53% |
| 20 | 1.49×10^-9^ A | 3.60×10^-4^ A | 84% | 54% |
| 30 | 4.63×10^-9^ A | 3.37×10^-4^ A | 59% | 52% |
| 40 | 2.97×10^-9^ A | 3.01×10^-4^ A | 66% | 44% |
| 50 | 1.44×10^-9^ A | 2.85×10^-4^ A | 60% | 40% |

**Table S2.** Figures-of-merit in typical perovskite photodetectors

| **Materials** | ***D****  **(cmW^-1^ Hz^1/2^)** | **Response Time** | | ***Wavelength* (nm)** | ***EQE*** | ***Ref*.** |
| --- | --- | --- | --- | --- | --- | --- |
|  |  | ***TPC* (ns)^a)^** | ***SSW* (μs) ^b)^** |  |  |  |
| CH_3_NH_3_PbI_3-x_Cl_x_ | - | - | 6.5/5.0 | 400-800 | 80% | 9 |
| MA_0.5_FA_0.5_Pb_0.5_Sn_0.5_I_3_ | 10^12^ | - | 7.4 | 400-940 | - | 10 |
| TiO_2_ nanorods/MAPbI_3_  heterojunction | 7.8×10^10^ | - | 10^6^ | 300-800 | - | 11 |
| MAPbI_3_ | 10^11^ | - | 2200/4000 | 265-800 | - | 12 |
| MAPbI_3_ single crystals | 1.5×10^13^ | 100 | - | 400-780 | 62% | 13 |
| PEIE/CsPbIBr_2_ | 9.7×10^12^ | 20 | - | 400-600 | 57.1%±3.1% | 14 |
| FA_0.85_Cs_0.15_PbI_3_ film | 2.7×10^13^ | 45/91 | - | 240-750 | - | 15 |
| OTPD/MAPbI_3_ | 7.4 ×10^12^ @680 nm | 120 |  | 300-780 | 80% | 1 |
| MAPbBr_3-x_I_x_ film | - | - | 20 | 300-550 | 55% | 16 |
| MoO_3_/MAPbI_3_ | - |  | 10 ± 0.8 | 300–780 | - | 17 |
| NiO_x_/MAPbI_3_/PCBM/PTB7-Th:IEICO-4F | ~10^10^ |  | 500/510 | 340–940 | 80%/70% | 18 |
| MAPbI_3_ | ~10^12^ | 0.95 |  | 400-800 | 80% | 19 |
| MAPbI_3_ nanowires | ~10^12^ | - | 200/300 | 390-850 | - | 20 |
| CsPbBr_3_ microcrystals | ~10^11^@0.1mw/mm^2^ | - | 500/1600 | 400-600 | - | 21 |
| CsBi_3_I_10_ thin films | ~2.5 × 10^12^ |  | 330/380 | 400-800 | - | 22 |
| **CH_3_NH_3_PbI_3_/ F8IC:PTB7-Th** | **>1×10^11^**  **380-780 nm**  **2.3×10^11^**  **@870 nm** | **5.6** | **35/20** | **300-1000** | **>63%**  **380-780 nm**  **54%@850 nm** | **This work** |

Here, we consider two test methods for response time.

1. Transient photocurrent method (TPC): The response time (also called decay time) is defined as the time for the photocurrent decrease from the peak to about 1/e after a single exponential fit for the TPC curve.
2. Standard square wave method (SSW): The rise/fall time is defined as the time for the photocurrent to increase/decrease from 10 %/90 % to 90 %/10 % of the peak when photocurrent arrives a stable state after accepting/removing illumination.

**SEM characterization**

**Fig. S7.** Cross-sectional SEM image of photodetectors. /Inset: perovskite film surface.

In this work, photodetectors based on the perovskite/organic bulk-heterojunction are composed of indium tin oxide (ITO)/poly(bis(4-phenyl)(2,4,6-trimethylphenyl)amine (PTAA)/CH_3_NH_3_PbI_3_ (MAPbI_3_)/F8IC:PTB7-Th (1:1)/C_60_/2,9-dimethyl-4,7-diphenyl-1,10-phenanthroline (BCP)/copper (Cu), and the thickness of different functional layer can be observed intuitively in the scanning electron microscope (SEM) sketch map. The perovskite (MAPbI_3_) crystals grew equably on the PTAA-modified ITO conductive glass, and organic BHJ (F8IC/PTB7-Th) acted as a NIR light photosensitive layer on perovskite UV-visible active layer.

**Application of photodetectors**

**
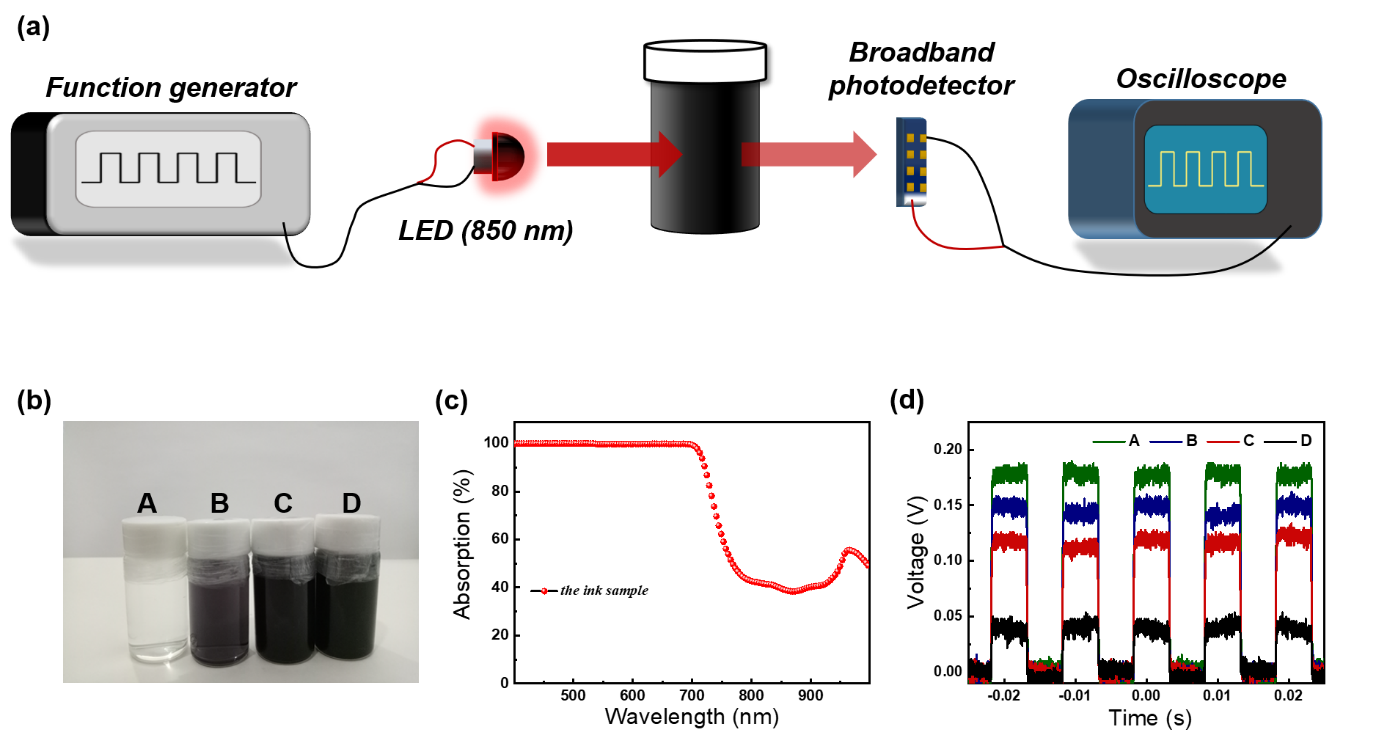
**

**Fig. S8.** a) Samples of solution mixed with different concentrations of ink. b) Different signal responses of samples A, B, C, D measured by the broadband photodetector and NIR LED light. c) The absorption spectrum of an ink sample. d) Schematic diagram of a simple water concentration measuring system.

To verify the performance of broadband photodetectors in practical application, we demonstrated a homemade detection system for measuring water purity. **Fig. S8a** is the schematic diagram of the measurement system. The purity of water is regulated by the concentration of the ink in it, where the turbidity of the ink in the sample is 0 ppm, 200 ppm, 1000 ppm, 10000 ppm, corresponding to A, B, C, D as shown in **Fig. S8b**. Here, the light emitter is a commercial LED with 850 nm emission peak, since visible light would be absorbed absolutely by the black sample as the absorption spectrum shown in **Fig. S8c**. It exhibited that the ink samples should be detected by partially absorbed infrared light rather than visible light, which would be completely absorbed so that no electrical signals could be generated. The working principle can be described as follow: A NIR LED driven by the function generators outputs periodic optical signal, which can effectively penetrate the sample. Then, the broadband photodetector can capture and display the NIR signal on the oscilloscope. Shown in **Fig. S8d,** as the ink concentration of the sample increases, the final output signals gradually decrease due to the NIR signal getting weaker and weaker. When the purity of the water of C and D is indistinguishable by the naked eye, the photodetectors enable a reasonable distinction without other analytical instruments. This design provides a new idea for the distinction of high concentration pollution detection of water purity.

More experimental details in the water purity application: As shown in **Fig. S8a**, the function generators drive the 850 nm LED with settled voltage and frequency to detect NIR optical signal. Because of the incomplete absorption in NIR wavelength by ink samples shown in **Fig. S8c** as follows, the NIR light passing through several ink samples will have a different degree of attenuation. It is the basic principle of the detecting process. The function generator provides voltage and frequency for LED so that the LED can emit a periodic NIR signal. After such a measurement system designing and building, the samples with different ink concentrations can be distinguished by the difference of the final output electrical signal of our photodetectors. On the other hand, the periodic output signal demonstrates the ability of our device to track frequency optical signals and excellent response-resilience. Besides, the dimension of the vial is 15 mm in diameter and a capacity of 3 ml.


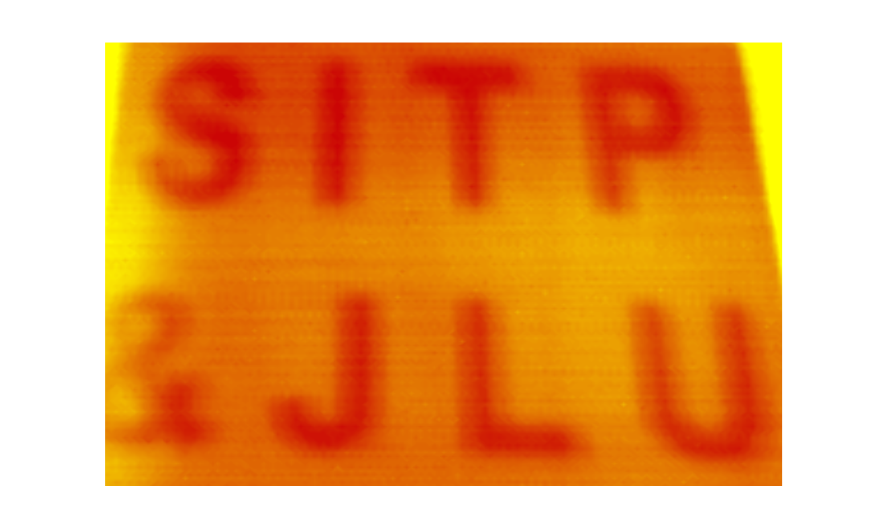


**Fig. S9.** Imaging of SITP&JLU letters detected by the perovskite/organic bulk-heterojunction photodetectors.

**Stability of photodetectors**

For the stability of our devices, the prepared broadband perovskite photodetectors were stored under extreme conditions, where the samples were exposed to air with more than 70% humidity and were heated on a 60 ℃ heating plate. The photoresponse and *EQE* of photodetectors were measured periodically to compare the falloff of stability in **Fig. S10**.


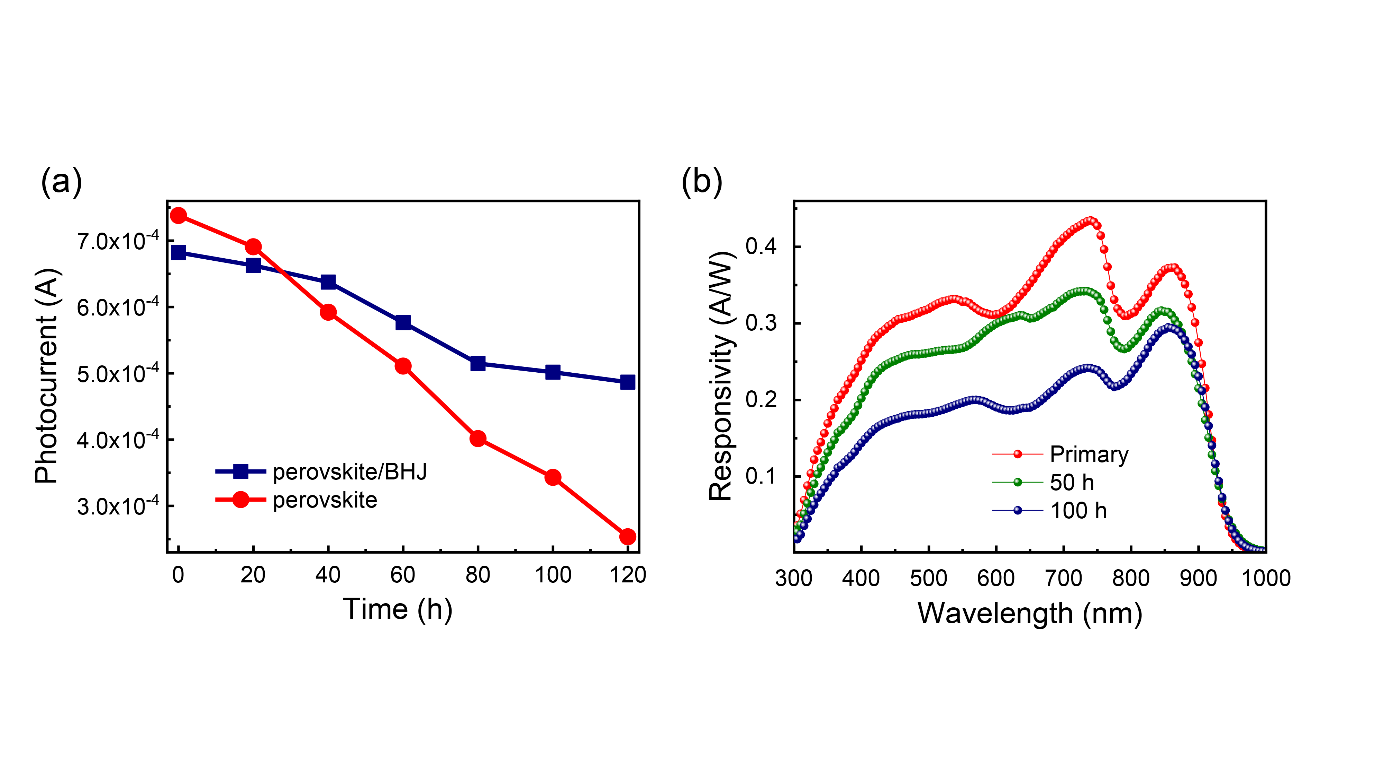


**Fig. S10.** Stability characterization of perovskite photodetectors. a) Photocurrent changes over time of perovskite/BHJ and pure perovskite devices under accelerated degradation conditions. b) Responsivity of perovskite/BHJ device over time under extreme conditions.

Under the condition of accelerated degradation for 120 hours, it can be found that the broadband photodetectors with BHJ have better stability than pure perovskite photodetectors, retaining more than 50% of the initial photocurrent under 0 V bias. Besides, as shown in **Fig. S10b**, after 100 h, the original device still maintained more than 50 % level of the primitive responsivity. This indicates that the perovskite photodetectors have good stability and can meet most application requirements under general conditions.

***IQE* of photodetectors**

The internal quantum efficiency (*IQE*) is indeed an important parameter for the new photodetector design/optimization. The *IQE* can be estimated by measuring absorbance and *EQE* curves. The formula for estimating *IQE* is *^23,24^*

$\text{IQE}\left( \text{λ} \right)\text{=}\frac{\text{EQE(λ)}}{\text{Abs}_{\text{AL}}\text{(λ)}}$

where *AL* holds for the active layer, and *Abs_AL_* is the absorption in the active layer.


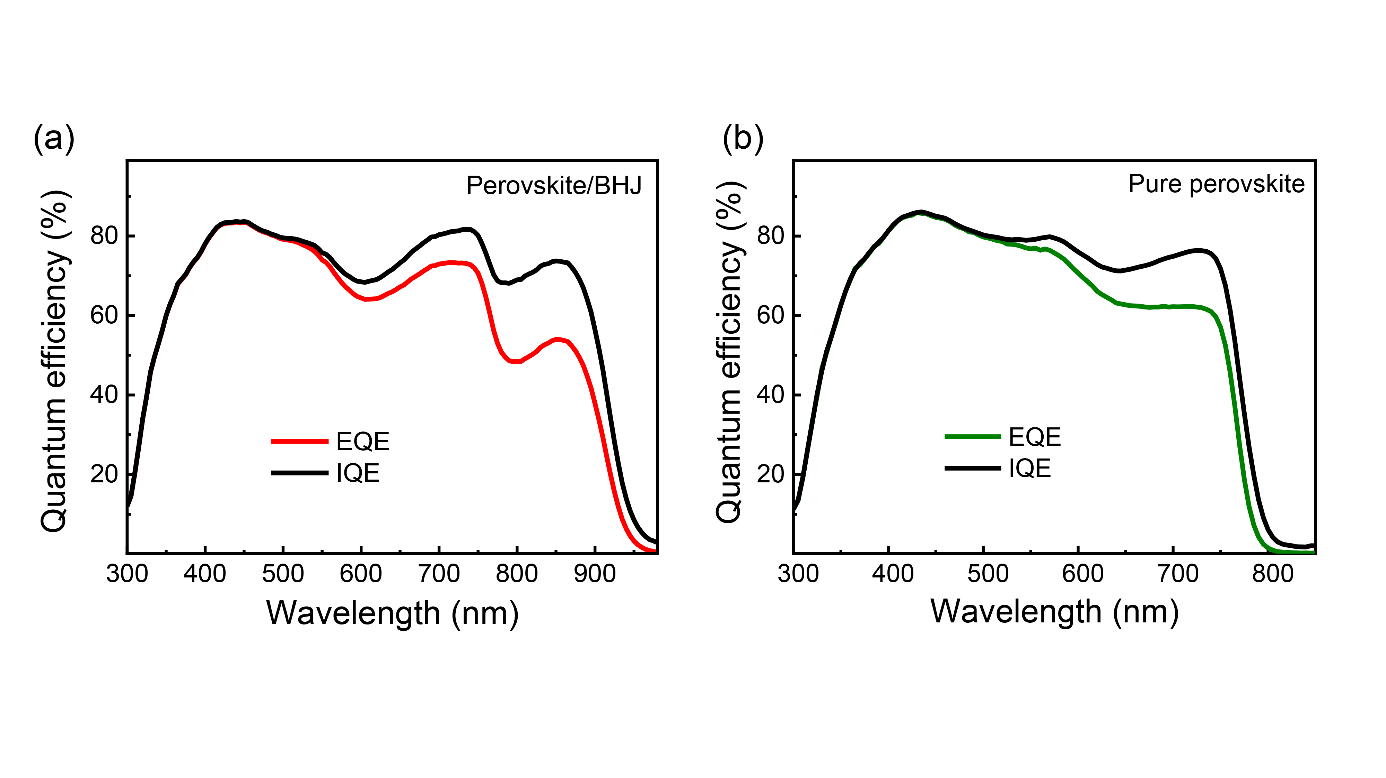


**Fig. S11.** *IQE* and *EQE* curves of perovskite/BHJ and pure perovskite devices.

As shown in **Fig. S11**, the theoretical value of *IQE* is always higher than the measured *EQE*. Obviously, the lack of the NIR region attributes to the absorption limitation of perovskite itself, and the introduction of organic BHJ absorption can solve this problem. The part of the curves where *IQE* is higher than *EQE* represents an effective absorption but not properly converted into an electrical signal, which is the carrier transport limited. This result shows that it may be possible to enhance carrier transport with other strategies to obtain higher *EQE* value, but the lack of NIR region results from the perovskite intrinsic absorption.

**References**

1. Fang, Y. J. & Huang, J. S. Resolving weak light of sub-picowatt per square centimeter by hybrid perovskite photodetectors enabled by noise reduction. *Advanced. Materials* **27**, 2804-2810 (2015).
2. Dong, Q. F. et al. Electron-hole diffusion lengths > 175 μm in solution-grown CH_3_NH_3_PbI_3_ single crystals. *Science* **347**, 967 (2015).
3. Castro-Carranza, A. et al. Effect of density of states on mobility in small-molecule n-Type organic thin-film transistors based on a perylene diimide. *IEEE Electron Device Letters* **33**, 1201 (2012).
4. Gao, H. Z. et al. Theoretical characterization of a typical hole/exciton-blocking material bathocuproine and its analogues. *Journal of Physical Chemistry A*  **112**, 9097 (2008).
5. Hutter, E. M. et al. Charge carriers in planar and meso-structured organic-inorganic perovskites: mobilities, lifetimes, and concentrations of trap states. *Journal of Physical Chemistry Letters* **6**, 3082 (2015).
6. Milot, R. L. et al. Temperature-dependent charge-carrier dynamics in CH_3_NH_3_PbI_3_ perovskite thin films. *Advanced Functional Materials* **25**, 6218-6227 (2015).
7. Reid, O. G. et al. Grain-size-limited mobility in methylammonium lead iodide perovskite thin films. *Acs Energy Letters* **1**, 561-565 (2016).
8. Kim, D. H. et al. 300% Enhancement of carrier mobility in uniaxial-oriented perovskite films formed by topotactic-oriented attachment. *Advanced Materials*  **29**, 1606831 (2017).
9. Li, F. et al. Ambipolar solution-processed hybrid perovskite phototransistors. *Nature Communications* **6**, 8238 (2015).
10. Xu, X. B. et al. High-performance near-IR photodetector using low-bandgap MA_0.5_FA_0.5_Pb_0.5_Sn_0.5_I_3_ perovskite. *Advanced Functional Materials* **27**, 1701053 (2017).
11. Zhou, H. et al. Self-powered, ultraviolet-visible perovskite photodetector based on TiO_2_ nanorods. *RSC advances* **6**, 6205 (2016).
12. Zheng, W. et al. Ultrafast-temporal-responsive flexible photodetector with high sensitivity based on high-crystallinity organic-inorganic perovskite nanoflakes. *Nanoscale* **9**, 12718-12726 (2017).
13. Bao, C. X. et al. Low-noise and large-linear-dynamic-range photodetectors based on hybrid-perovskite thin-single-crystals. *Advanced Materials* **29**, 1703209 (2017).
14. Bao, C. X. et al. High performance and stable all-inorganic metal halide perovskite-based photodetectors for optical communication applications. *Advanced Materials* **30**, 1803422 (2018).
15. Liang, F. X. et al. Broadband, ultrafast, self-driven photodetector based on cs-doped FAPbI_3_ perovskite thin film. *Advanced Optical Materials* **5**,1700654 (2017).
16. Wang, F. et al. Fast photoconductive responses in organometal halide perovskite photodetectors. *ACS Applied Materials & Interfaces* **8**, 2840 (2016).
17. Dong, R. et al. High-gain and low-driving-voltage photodetectors based on organolead triiodide perovskites. *Advanced Materials* **27**, 1912 (2015).
18. Wu, G. et al. Perovskite/organic bulk-heterojunction integrated ultrasensitive broadband photodetectors with high near-infrared external quantum efficiency over 70%. *Small* **14**, 1802349 (2018).
19. Shen, L. et al. A self-powered, sub-nanosecond-response solution-processed hybrid perovskite photodetector for time-resolved photoluminescence-lifetime detection. *Advanced Materials* **28**, 10794-10800 (2016).
20. Deng, H. et al. Growth, patterning and alignment of organolead iodide perovskite nanowires for optoelectronic devices. *Nanoscale* **7**, 4163 (2015).
21. Yang, B. et al. Ultrasensitive and fast all-inorganic perovskite-based photodetector via fast carrier diffusion. *Advanced Materials* **29**, 1703758 (2017).
22. Tong, X. W. et al. High-performance red-Light photodetector based on lead-free bismuth halide perovskite film. *ACS Applied Materials & Interfaces* **9**, 18977-18985 (2017).
23. Liang, Y. Y. et al. For the bright future-bulk heterojunction polymer solar cells with power conversion efficiency of 7.4%. *Advanced Materials* **22**, E135-E138 (2010).
24. Dennler, G. et al. Angle dependence of external and internal quantum efficiencies in bulk-heterojunction organic solar cells. *Journal of Applied Physics* **102**, 054516 (2017).
